# Beyond the Scope: Advancing Otolaryngology With Artificial Intelligence Integration

**DOI:** 10.7759/cureus.54248

**Published:** 2024-02-15

**Authors:** Bilal Irfan

**Affiliations:** 1 Microbiology and Immunology, University of Michigan, Ann Arbor, USA

**Keywords:** clinical practice integration, diagnostic precision, medical education, otolaryngology, artificial intelligence

## Abstract

The integration of artificial intelligence (AI) into otolaryngology heralds a new era of enhanced diagnostic precision, improved treatment strategies, and better patient outcomes. This advancement, however, brings to the fore the essential role of education and training in maximizing AI's potential within the field. The diverse spectrum of otolaryngology, encompassing audiology, rhinology, and sleep medicine, presents numerous opportunities for AI applications from predicting hearing loss progression and optimizing cochlear implant settings to managing chronic sinusitis and predicting the success of treatments for obstructive sleep apnea. Such innovations necessitate a paradigm shift in educational frameworks, merging traditional clinical skills with AI literacy. This involves introducing AI concepts, tools, and applications specific to otolaryngology in the curriculum, ensuring practitioners are equipped to leverage AI for diagnostics, patient monitoring, and surgical planning. Exploring the potential of large language models (LLMs) in medical education, simulating clinical scenarios for risk-free diagnostic practice and decision-making, is imperative. Underscoring the importance of continuous education for established otolaryngologists through workshops and seminars on the latest AI tools is another essential goal. Moreover, highlighting the need for a collaborative approach to address ethical considerations and ensure the responsible integration of AI while advocating for a multidisciplinary educational strategy is an important asset. As we navigate this transition, the commitment to training and education becomes paramount, preparing the otolaryngology community to embrace AI-driven healthcare innovations.

## Editorial

In the dynamic landscape of healthcare, otolaryngology stands at the precipice of a transformative era propelled by the integration of artificial intelligence (AI). The advent of AI in otolaryngology heralds a promise of unprecedented advancements, offering refined diagnostic precision, tailored treatment strategies, and enhanced patient outcomes. However, as we embark on this journey, the crux of realizing AI's full potential lies in addressing the pivotal aspect of training and education within the field.

Otolaryngology, with its broad spectrum encompassing disorders of the ear, nose, throat, head, and neck, presents a fertile ground for AI applications. From the nuanced differentiation of benign vs. malignant laryngeal lesions using AI-assisted narrow-band imaging to the precision of robotic surgeries for thyroidectomy, AI's impact is palpable across diverse otolaryngological practices [1]. Yet, the incorporation of AI into clinical workflows demands more than just technological readiness; it necessitates a profound shift in the educational paradigm for both current practitioners and the next generation of otolaryngologists.

AI's potential extends into more specific areas of otolaryngology, such as audiology, where algorithms could analyze audiometric data to predict hearing loss progression or optimize cochlear implant settings. Similarly, in rhinology, AI could revolutionize the management of chronic sinusitis by analyzing endoscopic images to predict disease flares or responses to therapy. In sleep medicine, machine learning models could predict outcomes of interventions for obstructive sleep apnea, including the likelihood of success with continuous positive airway pressure (CPAP) therapy or surgical interventions based on patient-specific characteristics.

The essence of integrating AI into otolaryngological practice hinges on a well-orchestrated educational framework that marries traditional clinical acumen with AI literacy. The current curriculum could evolve to introduce AI concepts, tools, and applications specific to otolaryngology, ensuring that trainees are adept at leveraging AI for diagnostic imaging, patient monitoring, and surgical planning. For instance, understanding the principles behind machine learning models used in predicting outcomes of cochlear implantations or the nuances of AI algorithms that enhance the detection of sinonasal pathology on CT scans is indispensable [2].

The integration of large language models (LLMs) into medical education offers a promising avenue to enrich learning experiences in otolaryngology. LLMs could simulate patient interactions or clinical scenarios, allowing students and residents to practice diagnostic reasoning and decision-making in a risk-free environment. These models can also provide immediate feedback, reinforcing learning and highlighting areas for improvement. Furthermore, LLMs could assist in the curation and summarization of the latest research findings, ensuring that otolaryngologists stay abreast of the rapidly evolving evidence base.

The rapid pace of AI advancements underscores the necessity for continuous education for established otolaryngologists. Workshops, seminars, and online courses focused on the latest AI tools, such as deep learning algorithms for classifying otitis media or AI systems for voice disorder assessments, should become a staple of professional development [3]. More research may need to be conducted to institute and curate guidelines for professionals. Furthermore, practical training on integrating AI-assisted diagnostic and therapeutic tools into everyday clinical practice will ensure that otolaryngologists remain at the forefront of patient care.

As AI becomes ingrained in otolaryngology, ethical considerations around patient consent, data privacy, and algorithmic bias must be integral to educational initiatives. Collaborative learning environments, where clinicians, AI researchers, and bioethicists engage in dialogue, can foster a comprehensive understanding of these critical issues. This multidisciplinary approach will not only enrich the learning experience but also pave the way for ethically responsible AI integration into clinical practice.

The integration of AI into otolaryngology presents an exhilarating yet challenging frontier. As we navigate this transition, the emphasis on training and education becomes paramount to avoid field-wide dependency. By fostering an AI-literate otolaryngology workforce, we can harness the full potential of AI to revolutionize patient care. With AI's proven capability to enhance diagnostic accuracy such as achieving a 90.91% accuracy in classifying laryngeal images as benign or malignant and in facilitating surgical precision, the future of otolaryngology, underpinned by AI, beckons a new era of innovation and excellence [1]. It is incumbent upon us, as members of the otolaryngological community, to embrace this change, ensuring that our training and education programs are as forward-thinking as the technology itself.

## References

[1] Hu R, Zhong Q, Xu ZG (2021). Application of deep convolutional neural networks in the diagnosis of laryngeal squamous cell carcinoma based on narrow band imaging endoscopy [Article in Chinese]. Zhonghua Er Bi Yan Hou Tou Jing Wai Ke Za Zhi.

[2] You E, Lin V, Mijovic T, Eskander A, Crowson MG (2020). Artificial intelligence applications in otology: a state of the art review. Otolaryngol Head Neck Surg.

[3] Ngombu S, Binol H, Gurcan MN, Moberly AC (2023). Advances in artificial intelligence to diagnose otitis media: state of the art review. Otolaryngol Head Neck Surg.

